# Developmental changes in Ca^2+^ signaling mediated by A_2A_ adenosine and mGluR_5_ glutamate receptors in olfactory bulb astrocytes

**DOI:** 10.1038/s41598-026-68163-9

**Published:** 2026-08-27

**Authors:** Fatemeh Mohammadpour, Antonia Beiersdorfer, Kiana Samad-Yazdtchi, Daniela Hirnet, Christian Lohr

**Affiliations:** 1 Division of Neurophysiology, University of Hamburg, Hamburg, Germany; 2 Center for Translational Neuromedicine, University of Copenhagen, Copenhagen, Denmark

**Keywords:** Astrocytes, Olfactory bulb, mGluR, Purinergic signaling, Development, Cell biology, Neuroscience

## Abstract

Astrocytes play pivotal roles in brain homeostasis and neuronal communication. Most astrocyte functions are orchestrated by intracellular Ca²⁺ signaling in response to neurotransmitters. Although astrocyte function has been widely studied, the ontogenetic development of their Ca²⁺ dynamics is not fully understood. This study investigated developmental changes in astrocyte Ca²⁺ signaling mediated by group I metabotropic glutamate receptors and adenosine receptors in the olfactory bulb. Stimulation with (R, S)-3,5-dihydroxyphenylglycine (DHPG), a group I mGluR agonist, evoked Ca^2+^ signals that were sensitive to the mGluR_5_ antagonist MTEP. Adenosine-evoked Ca^2+^ transients were inhibited by the A_2A_ antagonist ZM241385 in most astrocytes and by the A_2B_ antagonist PSB603 in a small number of astrocytes. Both the fraction of astrocytes that respond to stimulation of mGluR_5_ and A_2A/B_ receptors with Ca^2+^ responses and the amplitude of the responses significantly decreased during the first four weeks of postnatal development. Our results show that olfactory bulb astrocytes create glutamatergic and adenosinergic Ca²⁺ signaling that is downregulated as the olfactory bulb matures.

**Supplementary Information:**

The online version contains supplementary material available at https://doi.org/10.1038/s41598-026-68163-9.

## Introduction

**Fig. 1:**
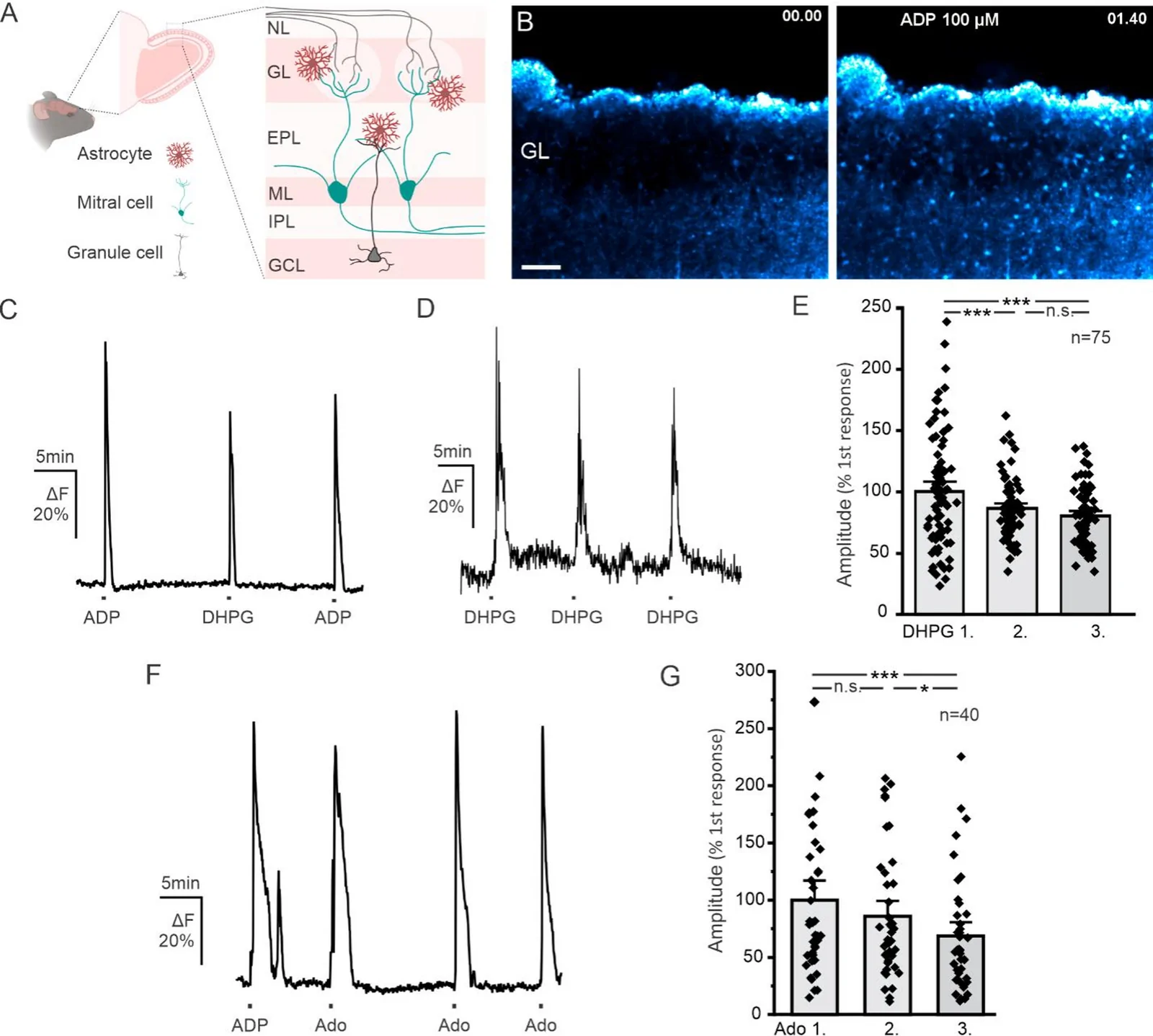
Activation of metabotropic glutamate receptors and adenosine receptors evokes Ca^2+^ signaling in olfactory bulb astrocytes. **A**) Schematic illustration of the mouse olfactory bulb and its distinct layers (NL = nerve layer; GL = glomerular layer; EPL = external plexiform layer; ML = mitral cell layer; IPL = internal plexiform layer; GCL = granule cell layer). **B**) Representative fluorescence images of the glomerular layer (GL) before and during bath application of ADP (100 µM). Scale bar: 50 μm. **C**) Example trace showing Ca²⁺ transients evoked by applications of ADP (100 µM, 30 s) and DHPG (100 µM, 30 s) and **D**) repetitive applications of DHPG (100 µM, 30 s). **E**) Quantification of DHPG-evoked Ca²⁺ amplitudes, expressed as a percentage of the mean first response. Significant reductions were observed between the first and second applications (***: *p* < 0.001) and between the first and third applications (***: *p* < 0.001). **F**) Example trace showing repetitive applications of adenosine (30 µM, 30 s). **G**) Quantification of adenosine-evoked Ca²⁺ responses across three applications, normalized to the first response. Significant differences were observed between the first and third responses (***: *p* < 0.001) and between the second and third responses (*: *p* < 0.05).

Astrocytes are abundant glial cells in the vertebrate central nervous system (CNS) that play crucial roles in supporting neural activity and maintaining brain homeostasis. Their excitation is reflected mostly by Ca^2+^ signaling with minor changes in the membrane potential, unlike neurons, whose excitation is conveyed primarily by electrical signals such as action potentials^1,2^. Ca^2+^ signals in astrocytes have multiple sources depending on the brain area and type of stimulation, reflecting their functional diversity^3,4^. As the primary processing center for olfactory information, the olfactory bulb (OB) receives input from sensory neurons in the nasal epithelium and transmits a processed and refined odor signal to higher-order brain areas such as the piriform cortex and amygdala. The OB comprises distinct layers, with sensory axons converging in the glomerular layer and forming synapses with mitral and tufted cells, the main projection neurons of the OB (Fig. 1A). These interact with interneurons in the glomerular layer and the external plexiform layer, modifying the input signals and shaping the olfactory information transmitted to the olfactory cortex^5,6^. Astrocytes contribute to the processing of sensory information in the OB via their response to neurotransmitters during neuronal activity, and inhibition of Ca^2+^ signaling in olfactory bulb astrocytes impairs odor discrimination and processing of stress-related conspecific odor cues^7–9^. Stimulation of olfactory sensory neuron axons in the olfactory bulb has been shown to result in the corelease of ATP and glutamate at their terminals in the glomerular layer^10^. Extracellular ATP is degraded to ADP and adenosine by ectonucleotidases such as CD39 and CD73, which in turn trigger an increase in intracellular Ca^2+^ in developing olfactory bulb astrocytes via P2Y_1_ and A_2A_ receptors^11^. In parallel, corelease of glutamate leads to Ca^2+^ influx through Ca^2+^-permeable AMPA receptors and internal Ca^2+^ release by the metabotropic glutamate receptor mGluR_5_^12–14^. Ca^2+^ signaling in astrocytes evoked by mGluR_5_ has been shown to be downregulated during early postnatal development of the mouse cortex and hippocampus^15^. Although pups are able to smell immediately after birth, the neuronal network of the olfactory bulb undergoes extensive rearrangement during the first three postnatal weeks. Crucially, these changes are not limited to neurons, but also involve the morphological refinement of astrocytes^16^. The application of mGluR_5_ agonists failed to stimulate significant Ca^2+^ signals via mGluR_5_ in adult olfactory bulb astrocytes^12,14^, raising the questions of whether mGluR_5_ is able to trigger Ca^2+^ signals in neonatal astrocytes and how this Ca^2+^ signaling develops postnatally.

In the present study, we investigated the development of Ca^2+^ signals evoked by glutamatergic and purinergic receptors in the astrocytes of acute mouse olfactory bulb slices during the first four postnatal weeks. The results revealed that during this time range, the fraction of astrocytes that respond to mGluR_5_ and A_2A_ activation with Ca^2+^ signals as well as the amplitude of the Ca^2+^ signals decreased.

## Results

### Adenosine and DHPG induce Ca²⁺ transients in olfactory bulb astrocytes

As illustrated in Fig. 1A, the olfactory bulb contains multiple layers, including the glomerular layer, where astrocytes and interneurons modulate the initial synaptic processing of odor signals. We focused our analyses on astrocytes located within this layer to assess age-dependent changes in Ca^2+^ signaling. In all experiments, we applied ADP (100 µM) to identify astrocytes and to verify the viability of astrocytes in the glomerular layer. In the age range investigated (P07-P26), ADP has been shown to reliably distinguish astrocytes from other cells in Fluo-8-loaded olfactory bulb slices, as olfactory bulb astrocytes express P2Y_1_ receptors and respond to purinergic stimulation with robust intracellular Ca²⁺ transients, whereas adjacent neurons remain unresponsive^11,12,17,18^. Since we have previously shown that astrocytes in the olfactory bulb of mice aged 7–10 days (P07–P10) respond to both ADP and adenosine with increases in Ca^2+^, we started our recordings at this age (Fig. 1B-C)^11^. We next assessed the effects of mGluR and adenosine receptor activation and whether repeated agonist exposure leads to desensitization of the receptors or the signaling cascade. We repetitively applied 100 µM DHPG (Fig. 1D-E), an agonist for group I metabotropic glutamate receptors comprising mGluR_1_ and mGluR_5_, and 30 µM adenosine (Fig. 1F-G) at 10-minute intervals, enabling us to track any potential reduction in Ca²⁺ transient amplitude over repetitive stimulations (rundown experiment). The first application of DHPG evoked a Ca²⁺ response with a mean amplitude of 82.8 ± 4.5% ΔF (*n* = 75). The second application produced a response that reached 86.4% of the initial amplitude and was significantly reduced (67.5 ± 3.3% ΔF; *n* = 75; *p* < 0.001). By the third application, the amplitudes significantly decreased to 81.9% of the initial response (64.1 ± 3.3% ΔF; *n* = 75; *p* < 0.001) (Fig. 1E). Notably, 8% of the DHPG-responsive cells (38 out of 475 cells, 16 brain slices, 12 animals) did not respond to ADP and hence were not identified as astrocytes. These cells, all located in the glomerular layer, presumably were neurons and were excluded from further analysis.

Upon the first application, adenosine (30 µM) evoked a Ca^2+^ response with an amplitude of 56.4 ± 6.4% ΔF (*n* = 40). The second application reached 86.0% of the initial response (48.4 ± 5.0% ΔF; *n* = 40) and was not significantly different. By the third application, the response significantly decreased to 68.7% of the first response (38.7 ± 4.4% ΔF; *n* = 40; *p* < 0.001) (Fig. 1G). All cells responding to adenosine also responded to ADP and were identified as astrocytes. The results show that both DHPG and adenosine evoke Ca^2+^ transients in olfactory bulb astrocytes with slightly decreasing amplitudes upon repetitive application. This might be due to desensitization of the receptors or incomplete refilling of the Ca^2+^ stores. In the following we compare amplitudes of Ca^2+^ transients in the presence of receptor antagonists with the values of the respective Ca^2+^ transient of these experiments and refer to them as “rundown control”.

### DHPG induced Ca^2+^ transients in olfactory bulb astrocytes predominantly via mGluR_5_

**Fig. 2:**
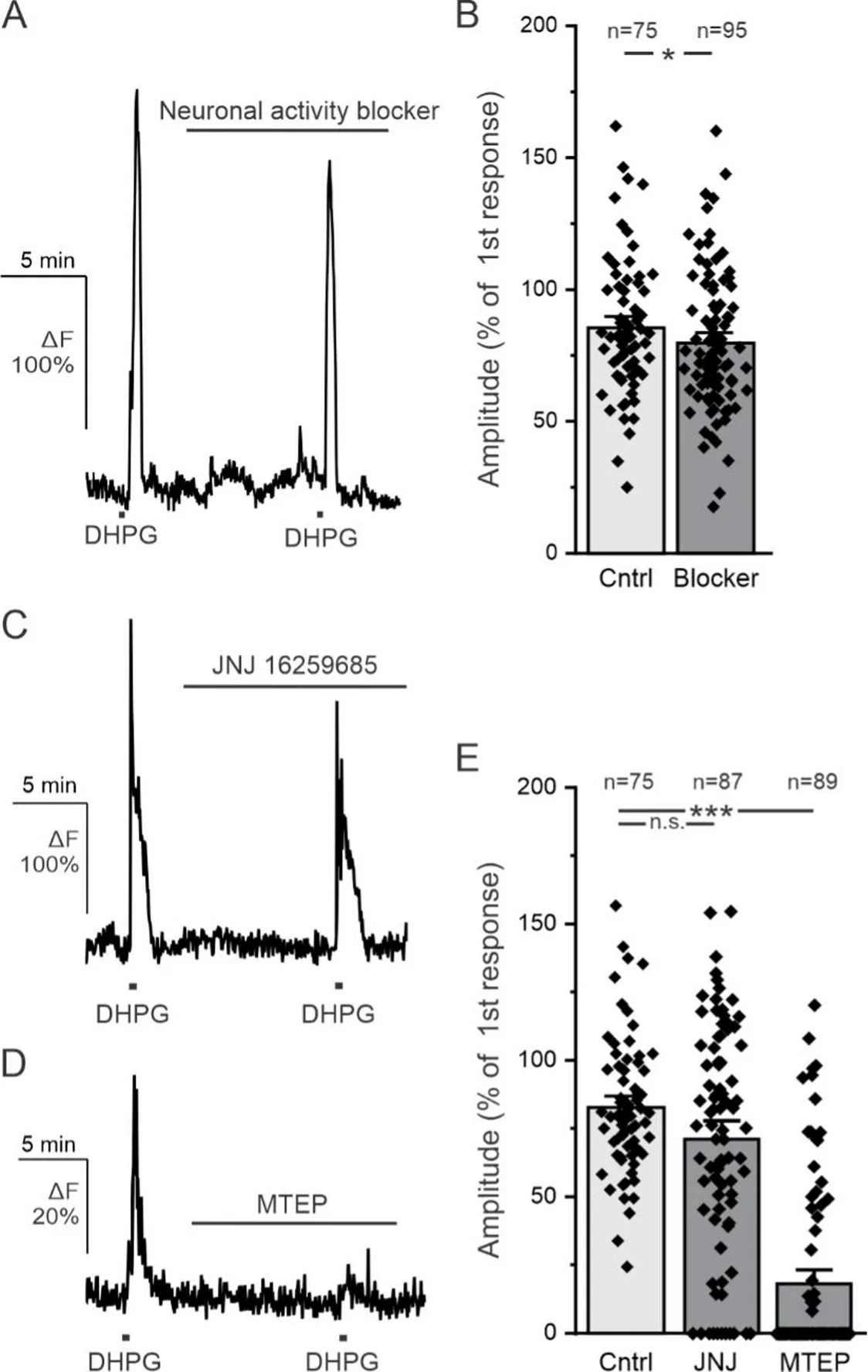
DHPG-evoked Ca^2+^ signaling is mediated by mGluR_5_. **A**) Representative Ca²⁺ traces showing DHPG-induced Ca^2+^ transients under control conditions and after the application of blockers (NBQX 10 µM, D-APV 50 µM, TTX 0.5 µM) to suppress neuronal activity. **B**) Quantification of DHPG-evoked Ca²⁺ amplitudes in the presence of the blocker mixture, expressed as a percentage of the mean first response, showing a small but significant reduction compared with the corresponding control in the rundown experiment (*: *p* < 0.05). **C**) Representative Ca²⁺ traces under control conditions and in the presence of a mGluR_1_ antagonist (JNJ 16259685, 200 nM) and **D**) a mGluR_5_ antagonist (MTEP, 10 µM). **E**) Bar graph showing the significant differences between DHPG-induced Ca²⁺ amplitudes under control conditions and in the presence of mGluR_5_ but not mGluR_1_ antagonists. (***: *p* < 0.001; n.s.: not significant).

Some neurons in the olfactory bulb express mGluR_1_ or mGluR_5_ and hence could be activated by DHPG, resulting in neurotransmitter release and possibly indirect Ca^2+^ signals in astrocytes^19,20^. To determine whether DHPG-induced Ca^2+^ signals in olfactory bulb astrocytes depend on neuronal activation, we tested the effects of tetrodotoxin (TTX) and ionotropic glutamate receptor antagonists on DHPG-evoked responses. TTX (0.5 µM) was applied to block action potentials, while the AMPA receptor antagonist NBQX (10 µM) and the NMDA receptor antagonist D-APV (50 µM) were used to suppress the involvement of ionotropic glutamate receptors in both neurons and astrocytes (Fig. 2A)^13^. Under these conditions, the amplitude of the DHPG-induced Ca²⁺ response averaged 78.2 ± 4.3% ΔF (*n* = 95) (Fig. 2B). This corresponds to 80% of the first response and was significantly smaller than the corresponding response in the rundown experiment (second application in Fig. 1E; *p* < 0.05), indicating that a small fraction of DHPG-evoked Ca^2+^ signaling in astrocytes is indirectly mediated by neuronal activity. Therefore, subsequent experiments were performed in the presence of TTX, NBQX, and D-APV to isolate the direct effect of the agonist on astrocytes.

We examined which metabotropic glutamate receptor subtype underlies DHPG-induced Ca²⁺ signaling in astrocytes. We focused on group I mGluRs and used the mGluR_1_ antagonist JNJ 16259685 (200 nM) and the mGluR_5_ antagonist MTEP (10 µM) (Fig. 2C-D). In the presence of JNJ 16259685, the amplitude of the DHPG-induced Ca²⁺ response reached 77.6 ± 6.2% ΔF (*n* = 87), corresponding to 71% of the first response, a difference that was not significantly different from the rundown control. In contrast, the application of MTEP reduced the DHPG-induced Ca²⁺ transient to 15.2 ± 3.2% ΔF (*n* = 89), corresponding to 18% of the control amplitude, a highly significant reduction (Fig. 2E; *p* < 0.001). Together, these results demonstrate that the largest part of the DHPG-evoked Ca²⁺ signal in olfactory bulb astrocytes is mediated by mGluR_5_.

### Adenosine-induced Ca²⁺ signaling in olfactory bulb astrocytes is mediated by A_2A_ receptors

**Fig. 3:**
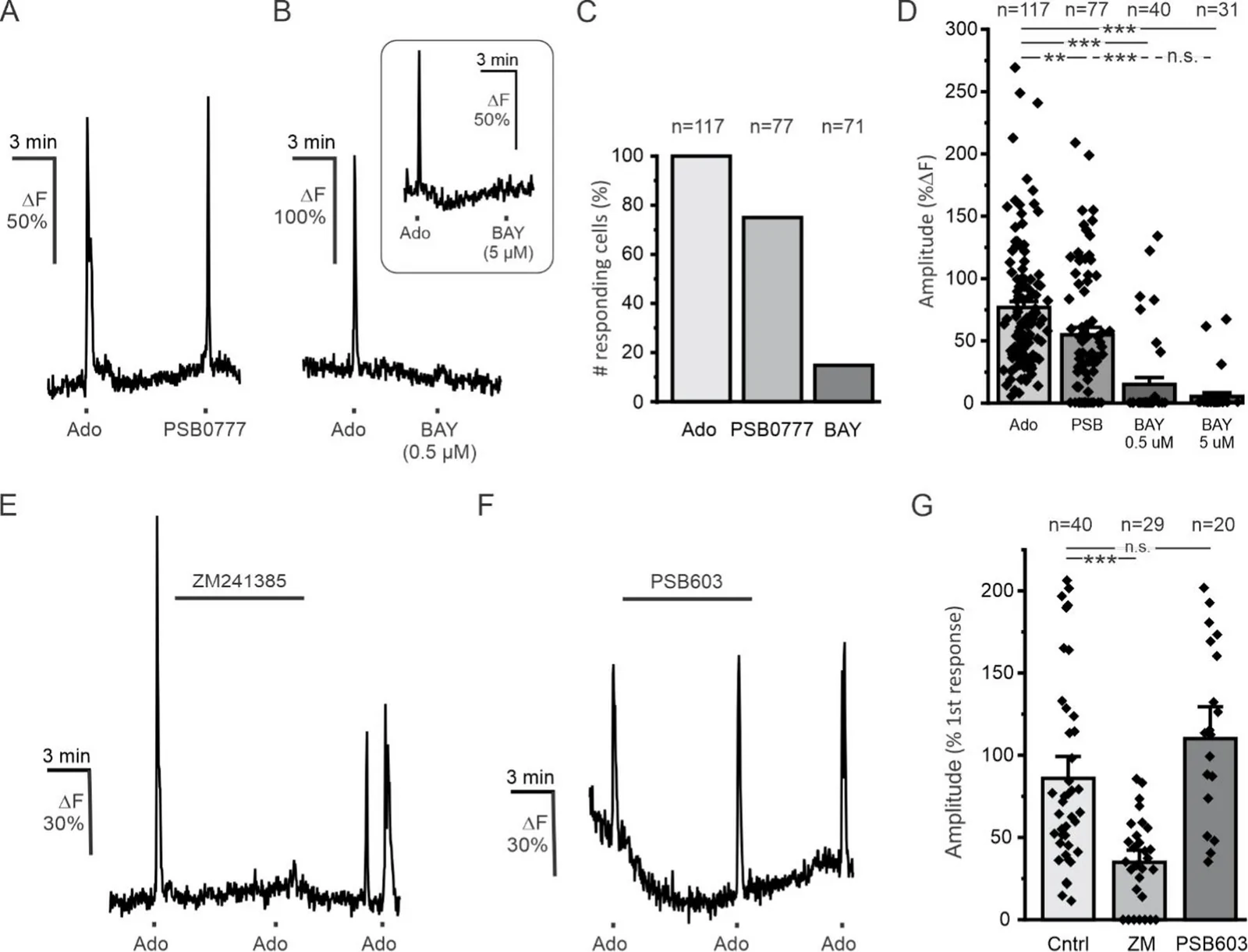
Adenosine evokes Ca^2+^ signaling via A_2A_ receptors. **A**) Representative Ca²⁺ trace showing the responses evoked by ADP (100 µM), adenosine (Ado, 30 µM) and the A_2A_ receptor agonist PSB0777 (5 µM) as well as **B**) the A_2B_ receptor agonist BAY 60-6583 (BAY, 0.5 µM). **C**) Proportion of astrocytes that responded to PSB0777 or BAY 60-6583, expressed as a percentage of the adenosine-responsive cell population, which was defined as 100%. **D**) Quantification of Ca²⁺ response amplitudes evoked by adenosine, PSB0777, and BAY 60-6583 (**: *p* < 0.01; ***: *p* < 0.001). **E**) Representative Ca²⁺ traces showing adenosine-evoked responses under control conditions and in the presence of the A_2A_ receptor antagonist ZM241385 (100 nM) and **F**) the A_2B_ receptor antagonist PSB603 (50 nM). The decrease in fluorescence during the first 5 min of the recording is due to a slight but recognizable movement of the tissue. **G**) Chart illustrating a significant reduction in adenosine-evoked Ca²⁺ amplitudes in the presence of the A_2A_ receptor antagonist ZM241385 (***: *p* < 0.001), whereas the A_2B_ receptor antagonist PSB603 had no significant effect (n.s.).

In developing olfactory bulb astrocytes, Ca^2+^ signals can be elicited by A_2A_ activation, whereas A_2B_ receptors mediate Ca^2+^ signaling in cortical astrocytes^11,21^. To identify which adenosine receptors mediate Ca²⁺ signaling in olfactory bulb astrocytes, we first applied selective agonists for A_2A_ and A_2B_ receptors. With respect to the cells that responded to adenosine, 75.3% (*n* = 77) also responded to the A_2A_ receptor agonist PSB0777 (5 µM), whereas only 15.5% (*n* = 71) responded to the A₂_B_ receptor agonist BAY 60-6583 (0.5–5.0 µM) (Fig. 3A-C). Both agonists elicited Ca^2+^ transients that were significantly smaller than adenosine-evoked Ca^2+^ transients (76.0 ± 4.7% ΔF; *n* = 117): PSB0777 induced robust Ca²⁺ transients with an amplitude of 54.3 ± 5.9% ΔF (*n* = 77; *p* < 0.001), whereas BAY 60-6583 elicited smaller responses of 34.8 ± 5.5% ΔF (*n* = 40; *p* < 0.001) at a concentration of 0.5 µM and 16.6 ± 3.0% ΔF (*n* = 31; *p* < 0.001) at a concentration of 5 µM (Fig. 3D).

To confirm the receptor subtype identification, we applied the A_2A_ antagonist ZM241385 (100 nM). In the presence of ZM241385, adenosine-induced Ca²⁺ responses were significantly reduced to 34.9 ± 4.9% of the amplitude of the first response (control; *n* = 29; *p* < 0.001). In contrast, the A_2B_ antagonist PSB603 (50 nM) did not significantly alter adenosine-evoked responses, which remained at 110.1 ± 12.9% of the first response (*n* = 20). Hence, adenosine triggers Ca^2+^ responses in developing olfactory bulb astrocytes mainly via A_2A_ receptors, confirming previous results^11^.

### Age-dependent decline in Ca²⁺ signaling evoked by DHPG and adenosine

**Fig. 4:**
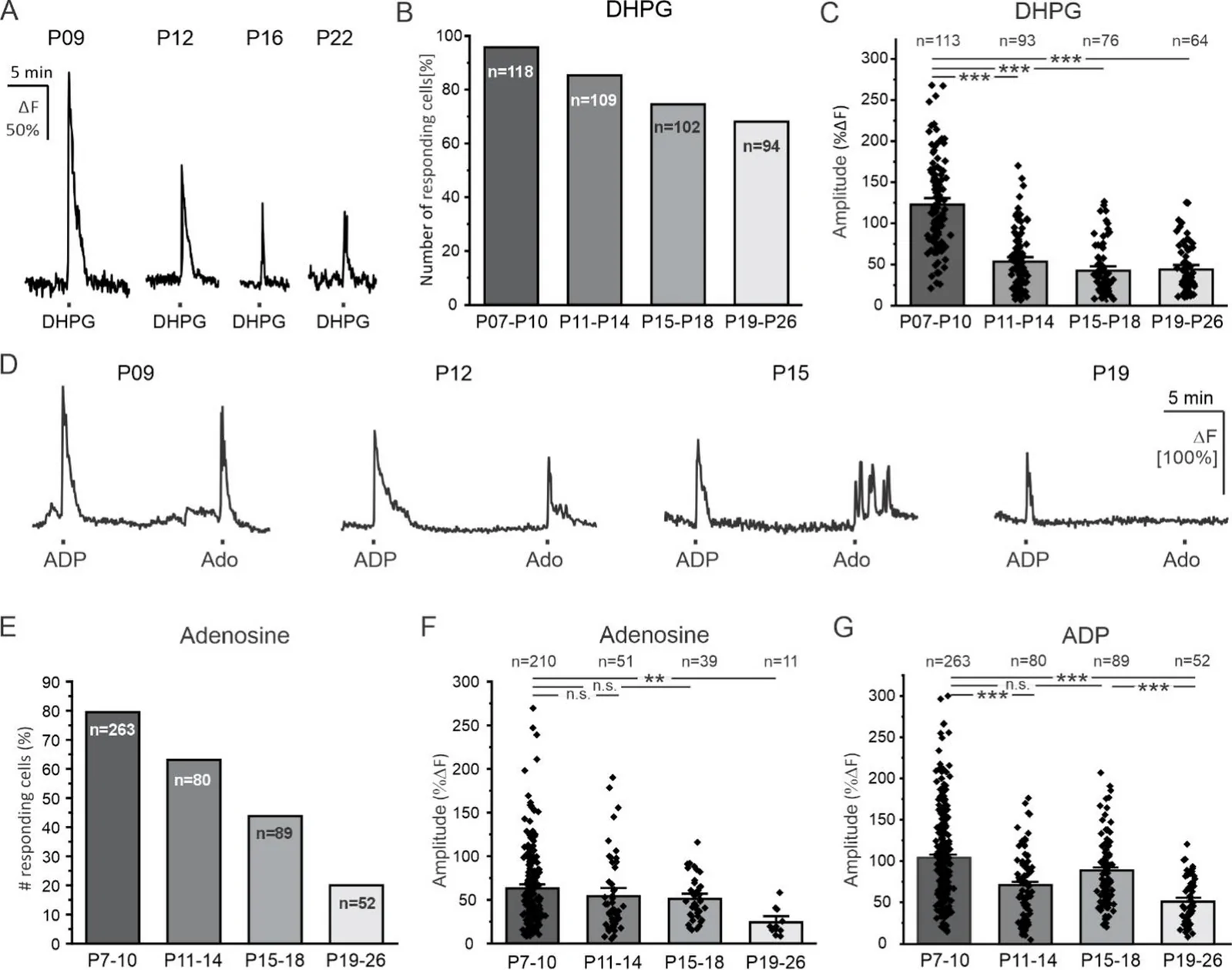
Development of Ca^2+^ signaling evoked by DHPG and adenosine. **A**) Representative Ca²⁺ traces showing DHPG-evoked responses (100 µM) in four developmental age groups, illustrating a progressive decrease in response amplitude with age. **B**) Percentage of astrocytes responding to DHPG across age groups, demonstrating a reduction in the proportion of responsive cells. **C**) Quantification of DHPG-evoked Ca²⁺ amplitudes across the four age groups. (***: *p* < 0.001). **D**) Example traces of Ca^2+^ increases evoked by ADP (100 µM) and adenosine (30 µM) in four different age groups. **E**) Graph depicting the decreasing number of astrocytes responsive to adenosine across four different age groups, showing a reduction in the number of responding cells from youngest to oldest. **F**) Quantification of adenosine-evoked Ca²⁺ amplitudes across ages (**: *p* < 0.01; n.s.: not significant). **G**) Quantification of ADP-evoked Ca²⁺ amplitudes across age groups (***: *p* < 0.001; n.s.: not significant).

Our results revealed that astrocytes in the developing olfactory bulb functionally express mGluR_5_. In astrocytes of the cortex and hippocampus, mGluR_5_ is strongly downregulated during the first three postnatal weeks^15^. Therefore, we analyzed DHPG-evoked Ca^2+^ transients in olfactory bulb astrocytes in four age groups, ranging from P07 to P26. All olfactory bulb astrocytes respond to ADP with Ca^2+^ signaling at that age, enabling the determination of the total number of astrocytes in a given experiment and the calculation of the fraction of astrocytes that respond to DHPG^11,17^. The fraction of DHPG-responsive astrocytes decreased with age (Fig. 4A and B). In the P07–P10 groups, 95.7% of the astrocytes (*n* = 118) responded to DHPG. This number declined to 85.3% in P11–P14 (*n* = 109), 74.5% in P15–P18 (*n* = 102), and 68.1% in P19–P26 (*n* = 94). The youngest group (P07–P10) presented the largest amplitudes, averaging 123.0 ± 5.2% ΔF (*n* = 113), whereas the older groups presented progressively lower mean amplitudes (P11–P14: 53.3 ± 3.7% ΔF, *n* = 93; P15–P18: 42.5 ± 3.5% ΔF; *n* = 76; P19–P26: 44.0 ± 3.7% ΔF; *n* = 64). Statistical comparisons revealed significant differences in the mean amplitudes of the DHPG-induced Ca²⁺ response among the first three age groups, whereas the amplitude did not decrease further between P15–18 and P19–26 (Fig. 4C).

In addition to the observed decrease in DHPG-evoked Ca²⁺ responses, we investigated age-dependent changes in Ca^2+^ signaling induced by adenosine and ADP in olfactory bulb astrocytes (Fig. 4D). Like the number of DHPG-responsive cells, the fraction of adenosine-responsive cells decreased with age (Fig. 4E). In the youngest group (P07–P10), 79.5% of the astrocytes responded to adenosine (*n* = 263). This number declined to 63.1% at P11–P14 (*n* = 80), 43.8% at P15–P18 (*n* = 89), and 20.0% at P19–P26 (*n* = 52), reflecting an age-dependent decrease in the fraction of adenosine-responsive astrocytes. The mean amplitude of adenosine-induced Ca²⁺ responses significantly decreased across developmental stages (Fig. 4F). In the P07–P10 groups, adenosine responses had a mean amplitude of 63.1 ± 3.1% ΔF (*n* = 210). This value progressively decreased in the older groups, with mean amplitudes of 54.2 ± 6.2% ΔF from P11–P14 (*n* = 51), 51.1 ± 3.9% ΔF from P15–P18 (*n* = 39), and 24.3 ± 4.7% ΔF from P19–P26 (*n* = 11). Statistical analysis revealed significant differences between the youngest and oldest groups (*p* < 0.01), indicating a reduction in adenosine-induced Ca^2+^ signaling during development.

During the time range investigated, astrocytes responded to ADP application with Ca^2+^ transients; however, we observed age-dependent changes in the amplitudes of ADP-evoked Ca^2+^ transients (Fig. 4G). The amplitude of the ADP responses was highest in the youngest group (P07–P10), with an average of 103.5 ± 3.8% ΔF (*n* = 263) and an average of 69.9 ± 4.7% ΔF in P11–P14 (*n* = 80), 87.6 ± 4.5% ΔF in P15–P18 (*n* = 89), and 51.5 ± 3.9% ΔF in P19–P26 (*n* = 52). Thus, ADP-induced Ca²⁺ responses slightly varied between P07 and P18 and considerably decreased after P18 (P19–P26; *p* < 0.001).

## Discussion

In this study, we studied astrocytes in the glomerular layer of the olfactory bulb, which were identified by their responsiveness to bath application of ADP. We have previously shown that astrocytes but not neurons generate Ca^2+^ responses to ADP in the olfactory bulb and cerebellum, rendering bath application of ADP a reliable means for astrocyte identification^11,17,18,22^. We characterized age-dependent changes in astrocytic Ca^2+^ signaling, with a focus on responses mediated by metabotropic glutamate and adenosine receptors. Ca^2+^ indicators with a single peak for each excitation and emission such as Fluo-8 do not allow for ratiometric measurements and hence do not provide information about the resting Ca^2+^ concentration in astrocytes, however, the amplitude of the fluorescence change reflects the amplitude of the Ca^2+^ increase and can be compared between different cells and age groups. Therefore, we only refer to the amplitude of the Ca^2+^ signals and not to differences in resting Ca^2+^ concentration between age groups. We found a steady, linear decrease in the number of astrocytes responding to DHPG between P07 and P26, while the amplitude of DHPG-evoked Ca^2+^ signals drastically dropped already between the first (P07-P10) and second (P11-P14) age groups. The age-related decrease in mGluR_5_-mediated astrocytic Ca^2+^ signaling observed in our study is consistent with previous findings demonstrating the developmental regulation of glutamatergic receptors in astrocytes, as a significant decrease in astrocytic mGluR_5_ receptors during postnatal development has been reported in the cortex and hippocampus^15^. In this study, a decrease in mGluR_5_ (*Grm5*) mRNA was detected in particular between the first and second postnatal week, indicating downregulation of expression. Ca^2+^ responses strongly decreased in amplitude during the same time window in our study, suggesting that this developmental change might be due to downregulation of *Grm5* expression. The dimension of the downregulation, however, appears to be less in the olfactory bulb, since in Sun et al.^15^*Grm5* was entirely downregulated within 4 weeks of postnatal development, while a fraction of astrocytes in the olfactory bulb in our study still responded to DHPG with Ca^2^^+^ transients. In the initial phase of postnatal development, astrocytic mGluR_5_ facilitates the detection of synaptically released glutamate, thereby inducing substantial Ca²⁺ responses. This process is imperative for the morphological and functional maturation of astrocytes. It involves the expansion of astrocytic domains and the induction of glutamate transporters^23^. As demonstrated by previous findings, the establishment of neuronal synapses is subject to prior interactions between neurons and astrocytes^24,25^. These findings suggest that astrocytic signaling plays a pivotal role in the process of synaptogenesis. The developmental downregulation of astrocytic mGluR_5_ presumably functions as a regulatory mechanism that restricts excessive synaptogenesis at the end of development and contributes to the stabilization of neural circuits by transitioning astrocytes to a mature state^26^.

In addition to mGluR_5_, adenosine receptors are involved in developmental regulation, and our results revealed a progressive decline in the peak amplitude of adenosine-induced Ca^2+^ transients and in the fraction of responding astrocytes across the four age groups examined. A study of the gene expression of cultured hypothalamic astrocytes revealed that the adenosine receptor profile changes with increasing age^27^. Compared with those in 90-day-old rats, the expression levels of A_1_ and A_3_ receptors in the astrocytes of newborn rats are lower. Conversely, A_2A_ receptor mRNA levels are higher in the older group^27^. However, our results show that adenosine-induced Ca^2+^ transients in developing astrocytes are predominantly mediated by adenosine A_2A_ receptors, with minimal contributions from A_2B_ receptors, and occur one week after birth, confirming previous results^11^. This apparent discrepancy from the study by Santos et al. (2024) may be indicative of several factors. First, the heterogeneity of astrocytes, both between species and across different brain regions, could be a contributing factor. Second, increased receptor expression does not necessarily lead to increased functional responses. This is due to age-related changes in G protein coupling efficiency or second messenger systems. Indeed, the decline in A_2A_-driven Ca^2+^ signaling is a consequence of a switch from a G_q_-coupled to a G_s_-coupled downstream pathway rather than a downregulation of A_2A_ expression since A_2A_ receptors are still functionally present in the olfactory bulb astrocytes of juvenile as well as adult mice and mediate cAMP signaling^28^. In summary, our results show that the repertoire of neurotransmitter receptor signaling in olfactory bulb astrocytes changes during the first postnatal week, which might mirror the neurochemical maturation of the olfactory bulb and the functional integration of astrocytes in neural circuits to determine neuronal performance and behavior^8,9^.

## Methods

### Animals

Olfactory bulb slices were prepared from C57BL/6 wild-type mice of both sexes. For developmental experiments, mice aged P7–P26 were used. The mice were bred and housed in the animal facilities of the Institute of Cell and Systems Biology, University of Hamburg, with a 12-h light/dark cycle and free access to food and water. All experiments were performed in accordance with the recommendations of the European Union and approved by the local ethics committee and authorities (Behörde für Justiz und Verbraucherschutz Hamburg; N010/2022). The ARRIVE guidelines for reporting the experiments were followed.

### Preparation of acute brain slices

The mice were anesthetized with 5% isoflurane in oxygen and then decapitated. The brains were dissected in chilled, Ca^2+^-reduced artificial cerebrospinal fluid (aCSF, see below) continuously gassed with carbogen (95% O₂/5% CO₂) and buffered to pH 7.4 throughout the preparation. Olfactory bulbs were removed and cut into 220 μm thick slices via a vibratome (Leica VT1200S, Bensheim, Germany). Slices were incubated in standard aCSF gassed with carbogen, initially at 30 °C for 30 min, and subsequently at room temperature until the experiments began.

### Reagents

The standard artificial cerebrospinal fluid (aCSF) used in all the experiments contained (in mM) 120 NaCl, 2.5 KCl, 2 CaCl₂, 1 MgCl₂, 2.8 D-glucose, 26 NaHCO₃, and 1 NaH₂PO₄ and was continuously bubbled with carbogen to maintain a pH of 7.4. A Ca^2+^-reduced aCSF was prepared with the following composition (in mM): 83 NaCl, 2.5 KCl, 0.5 CaCl₂, 2.5 MgSO₄, 20 D-glucose, 70 sucrose, 26.2 NaHCO₃, and 1 NaH₂PO₄. The group I mGluR agonist (R, S)-3,5-dihydroxyphenylglycine (DHPG) and the ionotropic NMDA glutamate receptor antagonist D-APV were obtained from Hello Bio (Dublin, Ireland). The mGluR_5_ antagonist MTEP hydrochloride, the mGluR_1_ antagonist JNJ 16259685, the adenosine A_2A_ agonist PSB0777 ammonium salt, the A_2B_ agonist BAY 60-6583, and the A_2B_ antagonist PSB603 were purchased from Tocris (Bristol, UK). The ionotropic AMPA glutamate receptor antagonist NBQX was obtained from Alomone Labs (Jerusalem, Israel), and adenosine diphosphate (ADP) was obtained from Applichem (Darmstadt, Germany). The A_2A_ antagonist ZM241385 was purchased from Abcam (Cambridge, UK), and the Na⁺ channel blocker tetrodotoxin (TTX) was supplied by Biozol (Hamburg, Germany). The Ca^2+^ indicator dye Fluo-8 AM was purchased from AAT Bioquest (Sunnyvale, CA, USA).

### Ca^2+^ imaging and data analysis

Acute brain slices were incubated with Fluo-8 AM (2 µM in aCSF) dissolved in DMSO and 20% pluronic acid for 40 min at room temperature. The brain slices were then placed in the recording chamber and continuously superfused with aCSF that was continuously gassed with carbogen. Drugs were applied via the same perfusion system. Image sequences were recorded at a rate of 3 s per frame via a confocal microscope (eC1, Nikon). The data were evaluated with EZ-C1 Viewer (Nikon), and statistical tests were performed with Origin Pro 10.1 (OriginLab Corporation, Northampton, USA). To analyze changes in the Ca^2+^ level in astrocytes, cells located in the glomerular layer were marked as regions of interest (ROIs) and the mean fluorescence intensity was measured throughout the image sequence. No background substraction was performed. The cells that showed a Ca^2+^ response to ADP were identified as astrocytes, since astrocytes but not neurons in Fluo-8-loaded olfactory bulb brain slices respond to ADP application with a fluorescence signal^11,12,17,18^. To evaluate changes in Ca^2+^ levels over time, fluorescence intensity (F) was recorded during the experiment and normalized to the basal fluorescence intensity in the absence of pharmacological stimuli. Changes in Ca^2+^ are given as changes in fluorescence (ΔF) as a percentage with respect to the basal fluorescence value immediately before the Ca^2+^ response, which was set as 100%. Ca^2+^ responses were assessed as amplitudes (baseline to peak). In experiments using receptor antagonists, the response in the presence of the antagonist was compared to the respective response of the “rundown” experiment performed without the antagonist to compensate for use-dependent, antagonist-independent attenuation of the response. All values are presented as the mean ± standard error of the mean with n representing the number of analyzed cells. Metadata such as the number of brain slices and animals used for each set of experiment are given in supplementary Table 1 (Suppl. Table 1). The proof of statistical significance was performed via Friedman ANOVA and the Wilcoxon post-hoc test for paired datasets at an error probability p (*: *p* < 0.05; **: *p* < 0.01; ***: *p* < 0.001). In the case of independent data, the Mann‒Whitney U test for two datasets and the Kruskal‒Wallis ANOVA with Dunn’s post hoc test for multiple datasets were applied. The Grubbs test was used to identify outliers throughout all the experiments, which were excluded from the analysis.

## Supplementary Information

Below is the link to the electronic supplementary material.


Supplementary Material 1


## Data Availability

The datasets generated during the current study are available from the corresponding author upon reasonable request.
