# Supplementary material for: Developmental changes in Ca2+ signaling mediated by A2A adenosine and mGluR5 glutamate receptors in olfactory bulb astrocytes
Source: Sci Rep. 2026 Aug 27;16:26939. doi: 10.1038/s41598-026-68163-9 (PMC13522448; doi:10.1038/s41598-026-68163-9)
Supplement: Supplementary file 1 [file 41598_2026_68163_MOESM1_ESM.pdf]

Developmental changes in Ca^{2+} signaling mediated by A_{2A} adenosine and $mGluR_5$ glutamate receptors in olfactory bulb astrocytes

Fatemeh Mohammadpour, Antonia Beiersdorfer, Kiana Samad-Yazdtchi, Daniela Hirnet, Christian Lohr

Supplementary table 1

Metadata of descriptive statistical analyses

Fig. 1 E

Application	Mean (ΔF%)	S.E.M.	# of cells	# of slices	# of animals
DHPG 1	82.8	4.5	75	3	3
DHPG 2	67.5	3.3	75	3	3
DHPG 3	64.1	3.3	75	3	3

Fig. 1 G

Application	Mean (ΔF%)	S.E.M.	# of cells	# of slices	# of animals
Ado 1	56.4	6.4	40	3	3
Ado 2	48.4	5.0	40	3	3
Ado 3	38.7	4.4	40	3	3

Fig. 2 B

Application	Mean (ΔF, % of 1st response)	S.E.M.	# of cells	# of slices	# of animals
DHPG control	86.4	2.7	75	3	3
DHPG Neuronal blocker	79.6	3.2	95	4	3

Fig. 2 E

Application	Mean (ΔF, % of 1st response)	S.E.M.	# of cells	# of slices	# of animals
DHPG control	86.4	2.7	75	3	3
DHPG + JNJ	71.2	4.4	87	4	3
DHPG + MTEP	18.1	3.4	89	4	3

Fig. 3 D

Application	Mean (ΔF%)	S.E.M.	# of cells	# of slices	# of animals
Ado	76.0	4.7	77	5	3
PSB0777	54.3	5.9	77	5	3
BAY 0.5 µM	34.8	5.5	40	4	3
BAY 5 µM	16.6	3.0	31	4	2

Fig. 3 G

Application	Mean (ΔF, % of 1st response)	S.E.M.	# of cells	# of slices	# of animals
Ado control	86.0	8.9	40	5	3
Ado + ZM	34.9	4.9	29	3	3
Ado + PSB603	110.1	12.9	20	3	3

Fig. 4 C

Application	Mean (ΔF%)	S.E.M.	# of cells	# of slices	# of animals
DHPG P07-10	123.0	5.2	113	3	3
DHPG P11-14	53.3	3.7	93	4	3
DHPG P15-18	42.5	3.5	76	4	3
DHPG P19-26	44.0	3.7	64	5	3

Fig. 4 F

Application	Mean (ΔF%)	S.E.M.	# of cells	# of slices	# of animals
Ado P07-10	63.1	3.1	210	7	3
Ado P11-14	54.2	6.2	51	3	3
Ado P15-18	51.1	3.9	39	5	3
Ado P19-26	24.3	4.7	11	5	3

Fig. 4 G

Application	Mean (ΔF%)	S.E.M.	# of cells	# of slices	# of animals
ADP P07-10	103.5	3.8	263	7	3
ADP P11-14	69.9	4.7	80	3	3
ADP P15-18	87.6	4.5	89	5	3
ADP P19-26	51.5	3.9	52	5	3
